# Movement Synchrony Over Time: What Is in the Trajectory of Dyadic Interactions in Workplace Coaching?

**DOI:** 10.3389/fpsyg.2022.845394

**Published:** 2022-04-28

**Authors:** Tünde Erdös, Paul Jansen

**Affiliations:** ^1^Department of Management and Organization, Amsterdam Business Research Institute (ABRI), Vrije Universiteit, Amsterdam, Netherlands; ^2^Department of Management & Organization, School of Business & Economics, Industrial Psychology, Vrije Universiteit Amsterdam, Amsterdam, Netherlands

**Keywords:** movement synchrony, non-verbal interactional processes, coaching process, demographics, number of sessions

## Abstract

**Background:**

Coaching is increasingly viewed as a dyadic exchange of verbal and non-verbal interactions driving clients' progress. Yet, little is known about how the trajectory of dyadic interactions plays out in workplace coaching.

**Method:**

This paper provides a multiple-step exploratory investigation of movement synchrony (MS) of dyads in workplace coaching. We analyzed a publicly available dataset of 173 video-taped dyads. Specifically, we averaged MS per session/dyad to explore the temporal patterns of MS across (a) the cluster of dyads that completed 10 sessions, and (b) a set of 173 dyadic interactions with a varied number of sessions. Additionally, we linked that pattern to several demographic predictors. The results indicate a differential downward trend of MS.

**Results:**

Demographic factors do not predict best fitting MS curve types, and only client age and coach experience show a small but significant correlation.

**Discussion:**

We provide contextualized interpretations of these findings and propose conceptual considerations and recommendations for future coaching process research and practice.

## Introduction

Today, there is increasing scholarly interest in exploring coaching as a set of verbal and non-verbal interactional processes in coach-client dyads (e.g., Schermuly and Scholl, 2012; Ianiro et al., 2013, 2015; Ianiro and Kauffeld, 2014; Erdös and Ramseyer, 2021). This interest is grounded in the nature of coaching as an interactional change process that is viewed as a “complex adaptive system” (O'Broin and Palmer, 2010, p. 28) rather than a linear input-output mechanism. Therefore, this paper answers calls from coaching scholars (e.g., Bachkirova, 2017; Myers, 2017; Erdös et al., 2020) to focus scientific efforts on exploring the impact of generic factors (i.e., dyadic interactional processes, time, and number of sessions) on the coaching process rather than specific techniques associated with any coaching method (Bachirova and Lawton Smith, 2015). Indeed, progressing the body of knowledge of generic factors on coaching may have the capacity to advance coaching as a purposeful meaning-making process (Drake, 2015). In that process, the present awareness of non-verbal aspects of the coach-client encounter is instrumentalized to explore dynamics in the coach-client relationship (Stelter, 2021) away from performance-driven outcomes toward sustained goal-directed behavior. This approach to progressing the body of knowledge in coaching characterizes what is currently referred to as third-generation coaching (Stelter, 2014). It is alleged to answer the challenges of a burnout society (Han, 2015) in which self-disciplining, as well as competing and controlling, for ever higher performance levels has become a trend toward disempowerment (Han, 2015).

Against this background, we know little about the role that the time and number of sessions as characteristic aspects of the trajectory of non-verbal interactions may play in a client's change process in coaching. Most recently, Erdös and Ramseyer (2021) have investigated the impact of movement synchrony (MS) as the unconscious non-verbal spontaneous responsiveness between a coach and a client on goal striving in workplace coaching. Specifically, the data analysis resulting from motion energy analysis (Ramseyer, 2020b) in that study produced a mean of the MS data by averaging MS cross-correlations between session segments and within dyads in sessions. In effect, that averaged data yielded differential findings, implying that coaching is a dynamic learning process with each session forming more than the sum of its individual parts. For instance, that study suggested three intriguing aspects of MS that warrant further inspection: (a) higher levels of MS were present at the outset of the coaching process, (b) MS showed a linear trend for a temporal decrease; and (c) a lower level of MS in a previous session predicted higher session-level outcomes in the next session. As that study did not differentiate between clusters of dyads or explore the temporal dynamics of MS across sessions, the question remains unanswered: what is in the trajectory of dyadic interactions? Answering this question may be important for deepening our understanding of the role of MS as a signal of effectiveness in coaching as a meaning-making process (Drake, 2015).

Apart from the rich albeit non-convergent evidence in psychotherapy literature (Ramseyer, 2020a) as a related field, there are few resources coaching researchers can draw on to understand the role of the temporal dynamics of MS in supporting a client's development through coaching. For instance, lack of convergence in therapy research has been shown in heterogenous associations between MS and process variables such as working alliance (Ramseyer, 2020a), and, rather, heterogenous results in association with therapeutic success (Paulick et al., 2018). Moreover, Lutz et al. (2020) indicated that MS in the third session resulted in lower success later in therapy, while other therapy research (Paulick et al., 2018; Schoenherr et al., 2019) revealed low MS to be an indicator of undesired drop-out and high MS to be a predictor of successful early termination of therapy. Apart from the richness of the most recent evidence based on MS in psychotherapy research, it is important to note that coaching is different from psychotherapy as a helping intervention in that coaching involves working with a non-clinical population (Peltier, 2011). Therefore, we are interested in building knowledge of MS as a time-series measure as a yet unexplored phenomenon in the coaching process. All the more, as the study by Erdös and Ramseyer (2021) indicated that MS may have a rich nature and multilayered facets for coaching as an interactional change process, hence, we claim that digging deeper into the serial representation of MS may be useful to yield insights that can be further investigated in coaching research on the basis of hypothesis-testing studies or in applying descriptive approaches to enhance our understanding of coaching as a change process.

Thus, this paper explores the relevance of the trajectory of MS in coach-client dyadic interactions based on the publicly available dataset from the study conducted by Erdös and Ramseyer (2021). It does so by computing average MS per session/dyad for two specific clusters: (a) those 59 dyads that completed exactly 10 sessions, and (b) a set of 173 dyads with a varied number of sessions. It depicts non-verbal interaction processes across sessions/dyads, providing best-fitting curves that represent the series of MS averages across sessions.

With this exploratory approach, this paper makes three contributions: it (1) complements the conceptual framework of MS in interactional processes in coaching, (2) integrates the shape of coaching dynamics across sessions as curve types of total coaching trajectories for two specific dyadic clusters, and (3) integrates demographic correlates of those curve types.

By our inductive approach, we hope to advance coaching theory and practice by providing a more integrated understanding of the possible dynamics of MS as a time-series measure, and the potential relevance of that dynamics for our meaning making of interactional processes across sessions in workplace coaching. It is by a more integrated approach to theory-driven research that coaching can move to become a more scientific and credible field of study (Bachkirova, 2017). We claim that coaching process research needs an integrated approach like the one proposed in this paper to provide a holistic theory-building design (Myers, 2017).

## Conceptual Background

### Movement Synchrony (MS)

Movement synchrony (MS; Ramseyer and Tschacher, 2011) is a yet unexplored concept in coaching. To date, the only study investigating MS as a spontaneous non-verbal interactional variable beyond consciousness in workplace coaching (Erdös and Ramseyer, 2021) found MS to have implications for clients' affective and cognitive regulatory capacities. That study also found MS to be influenced by the quality of the coach-client relationship in clients' goal-striving processes. Specifically, findings indicated that it is not “how much more” (i.e., quantity) but rather “how well” (i.e., quality) a coach and a client synchronize in sessions over time that may lead to clients regulating or dysregulating in coaching. In this vein, this paper considers MS as an operationalization of the psychological construct congruence (Shapiro, 1965) between a coach and a client in how well matched the coach and the client answer to each other through their non-verbal behavior. In social psychology, this “embodied congruence” was likewise found to be important for interactants' psychological safety (Feldman, 2007). Similarly, the level of embodied congruence (i.e., how well a coach and a client synchronize) may reflect changes in MS (i.e., increase or decrease) in the course of coaching, which this paper seeks to explore.

In principle, embodied congruence can reflect various phenomena. It can imply the level of transference and counter-transference dynamics (Freud, 1917) as the unconscious redirection of feelings from one person to the other in the coaching relationship (de Haan et al., 2011; Lee, 2014), or a non-verbal manifestation of enhanced listening in coaching (Whitworth et al., 2007). In one study (Turner, 2010), 83% out of 235 coaches reported considering non-verbal responsiveness as transference, while 77% reported considering non-verbal responsiveness as counter-transference. In another study (Cremona, 2010), coaches reflected on the physical manifestation of their emotional processes in the coaching process and gave feedback to clients about their own non-verbal responses as a way to cope with emotions. Current coaching research suggests various ways in which MS may serve as embodied contact to explore unconscious dynamics in coaching.

In their discussion, Erdös and Ramseyer (2021) interpreted MS as a “way of being present with clients authentically,” suggesting that the quality of non-verbal responses between a coach and a client (i.e., congruence) depends on how attentive the coach and the client are to each other's needs reciprocally. Therefore, we argue that it is important to start positioning MS theoretically as congruence in coaching literature. This unique positioning can enhance our understanding of the role of MS as a time-series measure rather than a quantified average and what it may predict or be associated with. Theoretically, congruence as a key element of the therapeutic relationship (Rogers, 1957) is well established in coaching (Jackson, 2017) and supports the factor of reciprocity of MS. However, being authentic with each other as an expression of reciprocal non-verbal exchanges has remained unexplored in coaching. So far, congruence has been investigated as a form of a coach's presence in coaching (Jackson, 2017). Specifically, presence has been looked at as the coach's capacity to direct awareness to the “here and now” of a coach-client interaction (Silsbee, 2008). Silsbee (2008) recognizes the relevance of a coach's internal congruence as “the body able to work in partnership” (Silsbee, 2008, p. 162) and recommends to work with somatic awareness, self-observation, and urges to catch conditioned responses. He claims that a coach's presence in coaching “evokes change in others” (Silsbee, 2008, p. 5). While coaching theory has so far focused on congruence as a within-person phenomenon, MS is conceived as a between-person interactional phenomenon. The relevance of MS as an authentic reciprocal accordance of non-verbal responses between a coach and a client is reflected in the dynamical systems view that action is followed by perception as conceptualized in ecological psychology (Gibson, 1966) and in phenomenological philosophy (Merleau-Ponty, 2002). In simple terms, the body provides authentic information that includes signals that will be used by interaction partners to navigate a social environment (Coey et al., 2012). This perspective follows Bluckert (2006) idea of the use of self: working with the awareness of cognitive and emotional responses in the body as the authentic instrument to indicate what is going on for us at the moment as a means to experience the client and to explore dynamics in the coach-client relationship. However, authentic embodied congruence has so far remained a coach-specific phenomenon rather than a concept of dyadic presence.

Conclusively, for the purposes of this paper and based on the findings by Erdös and Ramseyer (2021), MS reflects that both a coach and a client have needs in the coach-client relationship. Those needs may shape the way in which they respond to each other through MS in complex ways. Hence, we follow claims in coaching literature (Jackson, 2017) that physicality (i.e., body) is the instrument that indicates the extent to which the coach and the client are congruent at any given moment.

### Investigating the Trajectory of Coach-Client Dyads

First, some coaching researchers (Schermuly and Scholl, 2012; Ianiro et al., 2013, 2015; Ianiro and Kauffeld, 2014) have made first attempts to investigate interpersonal behavior, analyzing both the verbal and non-verbal behavioral exchanges act by coach-client dyads to understand interactional processes in coaching. One study (Ianiro and Kauffeld, 2014) comprising 48 coach-client dyads used the discussion coding system (DCS, Schermuly and Scholl, 2012) with four coders assessing affiliation and dominance expressions in a coach's and a client's interaction behavior. That study found a direct association between a coach's dominant-friendly coach behavior in the first session and (a) a client's dominant-friendly interaction behavior in that same session, and (b) the client's feeling safe after the third session in coaching. While dominant-friendly interaction behavior in micro-level session analysis does not reflect the essence of MS as a time-series measure across sessions, those studies share one core commonality: they investigated coaching as a process. The findings on interactional processes suggest that time and number of sessions play a key role in how clients progress through coaching.

Second, Erdös and Ramseyer (2021) call for coaching science to look beyond average levels of MS toward smaller-scale dynamics of MS, a phenomenon that is referred to as “symmetry building” and “symmetry breaking” (Boker et al., 2002). All the more, as low MS was associated with high goal attainment, high MS was associated with low goal attainment in that study. Boker et al. (2002) explored the nature of dyadic perception-action loops in posture and gesture during conversation to examine the temporal structure of symmetry formation and symmetry breaking between interactants involving the mirror system. The results of that study showed interactants to develop bias toward cyclic movement in mirror systems, that is, bias toward spatial mirror symmetry and temporal translation symmetry. Elsewhere, while some psychotherapy studies suggested a positive association between mean levels of non-verbal synchrony and various facets of therapy such as working alliance (e.g., Flückiger et al., 2018), those associations could not be corroborated across multiple sessions within dyads (Ramseyer et al., 2020).

These various developments call us to

- investigate the difference between mean levels of MS development and the specific temporal patterns and dynamics of MS across various numbers of sessions, and
- investigate the MS downward development (Erdös and Ramseyer, 2021), integrating more factors than reported so far (e.g., demographic and contextual factors including number of sessions) to clarify the role that several demographic factors may have in predicting MS as a time-series measure.

### Automated Measurement of Movement Synchrony

Applying automated measurement of publicly available video-based MS data (Erdös and Ramseyer, 2021), this study provides a more objective alternative to the use of coder ratings to measure non-verbal behavior in coaching process research (e.g., Bozer et al., 2013). While there are prominent examples of coding systems to measure non-verbal interactions such as the facial action coding system (Ekman et al., 2002) or the Berner system to assess body postures (Frey et al., 1982), these approaches are time-consuming and prone to error (Baesler and Burgoon, 1987).

The first studies that applied automated measurement of non-verbal synchrony investigated caregiver-infant interactions (Watanabe, 1983) in developmental research. Those studies found higher non-verbal synchrony in interactions between a caregiver and an own infant than in interactions with other infants (Bernieri et al., 1988). Since then, more generally, automated measurement of non-verbal synchrony in dyads in social psychology (Fujiwara et al., 2019) and psychotherapy (Paulick et al., 2018; Ramseyer, 2020a) has deepened our understanding of MS as the mutual regulation of dyadic meaning making (Tronick and andBeeghly, 2011). Today, we understand that interactional exchanges can represent alternating periods of dynamic patterns of matching, mismatching or reparation, which has implications for how relationships can develop over time.

To date, coaching research has remained focused on looking into effects of verbal rather than non-verbal behavior (e.g., Cilliers, 2005; Schermuly and Scholl, 2012; Bachkirova et al., 2015; Gessnitzer and Kauffeld, 2015). The few coaching studies on both verbal and non-verbal behavior (Ianiro et al., 2013, 2015; Ianiro and Kauffeld, 2014) have focused on the non-verbal behavior of either the client or the coach, showing, for instance, that the coach's non-verbal behavior plays a decisive role in the development of the coach-client relationship (Ianiro and Kauffeld, 2014). In effect, researching MS in coach-client dyads *via* automated measurement methods has remained unaddressed. This development may simply be due to the perception that we lack the availability of sufficiently objective and economic means of measurement rather than the lack in the interest in this research theme.

## Methods

### Design

First, this study was designed to enroll trained professional coaches specialized in various fields of coaching (i.e., leadership coaching, career management, business coaching) with adherence to at least one professional coaching organization. Several international professional coaching bodies (i.e., ICF, EMCC, WBECS, IoC) were involved in recruitment. Coaches were required to deliver workplace coaching in areas for day-to-day performance improvement and develop skill sets that would enable clients to take a proactive role in their workplace development.

Second, this study was designed to engage a maximally naturalistic sample (i.e., certified coaches, common clients, no students, no laboratory setting). The goal was to reflect the realities of coaching engagements as richly and authentically as possible (e.g., participants' choice of frequency and maximum duration of sessions, nature of contracting, coaching style, language used in coaching, the coaching method applied).

### Recruitment

First, for the purposes of statistical relevance, the goal was to recruit a minimum of 150 coach–client dyads. To enroll 150 dyads, the research project was presented at various international and national coaching, mentoring, and supervision conferences between 2017 and 2018. While the coaches were recruited directly by the corresponding author, the clients were recruited indirectly by the workplace coaches to ensure client anonymity.

Second, a rigorous pre-selection process was put in place to recruit coaches for this study. This process involved individual in-depth interviews with coaches conducted by the first author. The pre-selection interviews were clear about the requirements; the procedures of participation and the specific IT support provided for the purposes of this study. All the study-specific information shared in the recruitment interviews was made available transparently on a dedicated research website (www.coachingpresenceresearch.com). The research website also gave access to the detailed technical instructions for how to video-record sessions and how to transfer recorded files for the purposes of this study.

Third, following the pre-selection interviews, the coaches were granted a reflection period to decide their coming onboard. Recruitment was closed in January 2019. From originally 198 dyads indicating interest in participating in this research, 184 dyads eventually enrolled in this study and concluded their coaching processes successfully in line with the study requirements (Erdös and Ramseyer, 2021).

### Participants

First, Table 1 depicts the predominantly female gender distribution in this study. Table 2 depicts more or less mixed age characteristics, as could be expected from the design selected for this study.

**Table 1:** Overview of demographic factors as predictors *N* = 59.

***N* = 59**	**Factors**	**Coach**	**Client**	**Same gender pair**
	Male	11	20	8
	Female	48	39	36
	Mixed gender pairs			15
	Below age 26		4	
	Age group 26–45		34	
	Age group 46–60		15	
	Above age 60		6	

*N = depicts the number of dyads with 10 coaching sessions*.

**Table 2:** Overview of demographic factors as predictors, *N* = 173.

***N* = 173**	**Factors**	**Coach**	**Client**	**Same gender pair**
	Male	29	144	14
	Female	48	112	95
	Mixed gender pairs			64
	Below age 26		9	
	Age group 26–45		98	
	Age group 36–60		56	
	Above age 60		9	
	Invalid age bracket		1	
	Coach experience y = 1–9	81		
	Coach experience y = 10+	82		
	Coach experience y = 16+	10		

*N = depicts the number of dyads with a varied number of coaching sessions. y = depicts the number of years of experience that coaches had on engaging in the research project*.

Second, coach participation was based on the coach's level of experience rather than the coach age, which was defined by three categories: 1–9 years (*N* = 49 coaches), 10 + years (*N* = 46 coaches), and 16 + years (*N* = 5 coaches) for the purposes of this study.

Third, this study design allowed for coaches to work with several clients: *N* = 32 coaches engaged with *n* = 1 client, *N* = 55 coaches engaged with *n* = 2 clients, *N* = 8 coaches engaged with *n* = 3 clients, and *N* = 5 coaches engaged with *n* = 4 clients.

Fourth, for the purposes of automated video-data analysis in coaching as a non-clinical helping intervention, it was assumed that clients have the full capacity to non-verbally synchronize (e.g., Ramseyer and Tschacher, 2011) and are not influenced by diagnostically relevant criteria (e.g., psychosis, substance dependency).

### Data Collection

**Figure 1:**
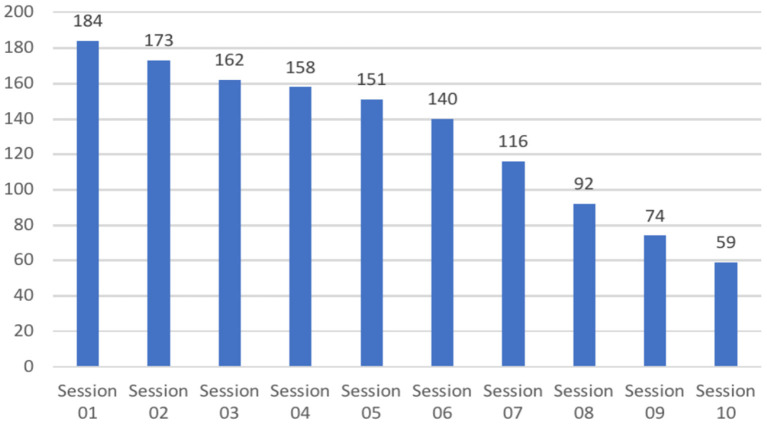
Frequency distribution of sessions in the data collection phase. Data collection phase lasted from October 2018 through to November 2019. 184 dyads completed one session; 59 dyads completed 10 sessions.

First, this study was designed to comprise up to a maximum of 10 dyadic interventions (i.e., sessions), each with minimum duration of 60 min (i.e., video segments), which is standard contracting practice in coaching. In terms of the number of sessions, each dyad was free to contract the specific number of sessions appropriate for their coaching process. For instance, Figure 1 depicts the number of dyads that completed at least 7 sessions (*N* = 116 dyadic interventions) or those dyads that completed 10 sessions (*N* = 59 dyadic interventions). For the purposes of this study, Figure 1 (from Session 2 to Session 10) provides an overview of the entire set of *n* = 1,309 sessions that were completed by all the dyads participating in this study.

Second, coaches video-recorded each coaching session in the naturalistic setting of their coaching engagement. The goal was to capture real-time face-to-face interactions through body movement for further MS analysis. Video recordings were conducted in line with key specifications (see www.psync.ch for details) defined for successful data analysis with motion energy analysis (MEA; Ramseyer, 2020b). Video data were collected and video files were transmitted in compliance with general data protection regulations (GDPR). The research project was awarded an ethics approval by the authors' research institute. All study participants had signed a written informed-consent form prior to their enrollment in this study.

Fourth, the entire data set comprises *N* = 173 out of *N* = 184 coach-client dyads with a set of *n* = 982 sessions (Figure 1) in this study. *N* = 11 dyads had only one session and were excluded from the research sample as no change from session to session can be shown for these dyads.

### Motion Energy Analysis

MEA (Version 4.03; see www.psync.ch) was selected as the approach to measure MS in this study (Erdös and Ramseyer, 2021). Analyzing the first 25 min of each session, this specific automated approach allowed for maximum comparability across different recording settings (Ramseyer and Tschacher, 2011). Furthermore, the MEA approach also required the thorough visual inspection of videos for potential anomalies (e.g., lighting, video resolution, etc.) as a variety of video recording devices (i.e., iPhone, camcorder, PC) were used for recording purposes in this study. Originally, in screening a total of *N* = 1,323 video files, *n* = 14 files were excluded as low video quality could have produced erroneous analytical results.

The R-package rMEA (Kleinbub and Ramseyer, 2020) was selected as the statistical approach to calculate an index of MS (i.e., the coordination of movements between a coach and a client). Time series of each initial 25-min dyadic interaction were cross-correlated to obtain MS values for the simultaneous and time-lagged coordination of a coach's and a client's body movement (function MEAccf in rMEA). Time-lagged movement coordination was correlated based on ± 5 s (lagsec = 5). That is, each ± 5 s of coach-client movement was coded as a cross-correlated coefficient. Subsequently, the time-lagged correlation coefficients were computed to Fisher's Z values before averaging those values to a grand mean of MS per session per 25-min session segment (r2Z = T; ABS = T). Eventually, the overall MS index is the absolute grand mean of cross-correlation values over all 25-min segments across all sessions of each coach-client trajectory (Ramseyer, 2020b).

### Exploratory Approach

As the goal in this study was to investigate the temporal dynamics of MS in the coaching trajectory, the exploratory approach selected was curve fitting. Curves can be computed based on the average MS per session across a range of sessions (i.e., 2,.., 10), that is the total coaching trajectory. This approach allows for investigating trends in average dyadic MS in a total coaching trajectory. Curve fitting involves classifying types of MS patterns per dyad and number of sessions on the basis of the general shape of the curve, and allows for computing curve parameters (i.e., linear increase/linear decrease, quadratic, cubic). Curve fitting can be used for further statistical analyses (i.e., regression analyses). As such, it is one of the most powerful and widely used analytical tools to examine the relationship between one or more predictors and a multiple (e.g., multiple time points) response variable. Thus, our aim was to compute a “best fit” model of such a relationship based on the varying number of sessions per dyad (Figure 1).

We used curve estimation as a statistical approach to explore a number of curve types that would best depict MS patterns of dyads across sessions. Curve estimation was conducted in two iterations for: (i) 59 dyads (590 dyadic interactions, 10 sessions) and (ii) 173 dyads (982 dyadic interactions computed as follows: 173^*^2 + 162 −158 + 151 + 140 + 116 + 92 + 74 + 59 = 982). First, this approach was found to be most appropriate to depict the longest time series available in the dataset to answer the two research questions. The reason is that the curve part representing max. 5 sessions is based on the biggest amount of synchrony data per dyad available in the dataset. That curve part depicts more data points than the curve part representing 10 sessions. Second, we aimed to explore the possible impact of demographic factors such as client age, coach gender, client gender, coach years of experience, as well as number of sessions on the curve type identified to best fit the MS pattern per dyad over time (*N* = 173).

### Data Analysis

After plotting average session MS per dyad and the number of sessions completed per dyad, data were transferred to SPSS V.23. The curve estimation procedure was selected to produce curve estimation regression statistics and related plots for 8 different curve estimation regression models (i.e., cubic, exponential, linear, quadratic, inverse, logarithmic, growth, and S-curve) as these models were expected to show the best curve fit for each dyad. For each model, ANOVA produced values for regression coefficients, multiple R, R2, adjusted R2, standard error of the estimate, predicted values, residuals, and t-statistics as prediction intervals. The model also showed F-statistics to account for the improvement in model error as well as the *p*-value to further show predictive significance in the model. Specifically, the best curve fit was determined for each curve plot using R2/F/and *p*-value as effects of significance and visually inspected by a researcher and a statistician applying the four-eye principle. As all-lag values for MS are usually small in rMEA (e.g., for dyad 101, session 1, all-lag value equals 0.1521), data were increased by Factor 10 (i.e.,0.1521 becomes 1.521) for calculating the estimated best curve fit. This factoring step was carried out to improve the readability of data and did not affect statistical results.

First, the curve fitting process resulted in the reduction of curve types to the most commonly occurring ones to comprise the cubic/quadratic/linear decrease/linear increase/linear constant curve types. We excluded curve types with no or few entries in that particular data sample using the curve estimation equations:

Linear model equation is Y = b0 + b1 ^*^ t.

Quadratic model equation is Y = b0 + b1 ^*^ t + b2 ^*^ t^2^.

Cubic model equation is Y = b0 + b1 ^*^ t + b2 ^*^ t^2^ + b3 ^*^ t^3^,

where Y is MS, bx (x = 0, …, 3) is regression coefficients, and t represent time series (i.e., 1 to 10).

Second, as the 59 dyads that completed 10 sessions (i.e., 590 interactions) provided the highest resolution for curve fit, regression estimation was rerun to estimate best curve fits using the reduced curve types across those dyads again. For an overview of best-curve-fit estimations, mean session MS values were used for all dyads that completed 10 sessions to calculate the best curve fit for linear/cubic/quadratic curve types. About 11 of the 32 dyads with the best fit for linear curvature had a significant linear fit (*p* < 0.05). In all those significant cases, b was negative. About 5 of the 27 dyads with the best fit for non-linear curvature had a significant non-linear fit; specifically, we found a significant linear fit for 11 dyads, a significant quadratic fit for 4 dyads, and a significant cubic fit for 1 dyad.

Third, we repeated the same data analysis process per each dyadic interaction (*N* = 173) and the varied number of sessions in the dataset. About 20 of the 79 dyads with the best fit for linear curvature had a significant linear fit (*p* < 0.05). In all those significant cases, b was negative. About 17 of the 94 dyads with the best fit for non-linear curvature had a significant non-linear fit; specifically, we found a significant linear fit for 17 dyads, a significant quadratic fit for 10 dyads, and a significant cubic fit for 7 dyads.

Finally, the (linear, quadratic, and cubic) b values for each dyad curve (*N* = 173) were regressed on the demographic factors as predictors. Combining all data in one regression analysis to identify the predictive value for various MS pattern curve types [i.e., linear in/decrease (b), quadratic (b^2^), cubic (b^3^), etc.] which allowed for a more sensitive approach than selecting general curve types as the dependent variable. Each dyad includes b, b^2^, and b^3^ value entries and demographic predictors for all participants irrespective of the level of significance of these values.

## Results

### Statistical Results

As reported by Erdös and Ramseyer (2021), observed synchrony was found to be significantly different from coincidental synchrony [*t*_(382.8)_ = 9.10; *p* < 0.001]. The difference between observed and coincidental synchrony had a medium effect size (Cohen's d = 0.67). Across all subjects, synchrony decreased over time (session = −0.001; *t*_(1,161.2)_ = −4.09; *p* < 0.001; ICC = 0.625).

### Exploratory Outcomes

#### Best Fitting Curves for Dyads With 10 Coaching Sessions

Supplementary Table 1 shows the entire set of best curve fit estimation values for all the 32 dyads in the cluster identified with the best “linear” (decrease, increase, or constant) curve fit. Supplementary Table 2 shows the entire set of best curve fit estimation values for all the dyads (*n* = 27) in the cluster identified with the best “cubic” and “quadratic” curve fit. Subsequent visual inspection of the curve plot confirmed the best fitting curves.

For an overview, Table 3 depicts the mean best curve fit estimation value for all the dyads in the cluster identified with the best “linear” curve fit (*n* = 32), as well as for all the dyads in the cluster identified with the best “cubic” curve fit (*n* = 14) and “quadratic” curve fit (*n* = 13).

**Table 3:** Mean linear and non-linear curve fit—dyads with 10 sessions.

	**Mean linear and non-linear curve fit—dyads with 10 sessions**														
	**Model Summary**				**ANOVA**						**Coefficients**				
											**Unstandardized coefficients**		**Standardized coefficients**		
**Curve**	**R Square**	**Adj R Square**	**Std. Error of Est**.		**Sum of squares**	**df**	**Mean Square**	**F**	**Sig./** ***p*** **-value**		**B**	**Coeff. St. Error**	**Beta**	***t***	**Sig**.
Linear	0.320	0.219	0.119	Regression	0.104	1	0.104	8.098	0.286	Session	−0.021	0.014	−0.333	−1.613	0.286
				Residual	0.143	8	0.018			(Constant)	1.341	0.087		16.834	0.000
															
Quadratic	0.428	0.264	0.119	Regression	0.138	2	0.069	3.284	0.205	Session	−0.052	0.059	−0.866	−0.719	0.137
				Residual	0.119	7	0.017			Session** 2	0.005	0.005	0.894	0.754	0.121
										(Constant)	1.384	0.140		11.634	0.000
															
Cubic	0.342	0.013	0.144	Regression	0.083	3	0.028	1.508	0.496	Session	−0.025	0.210	−0.681	−0.242	0.307
				Residual	0.134	6	0.022			Session**2	0.002	0.043	1.032	0.170	0.277
										Session**3	0.000	0.003	−0.380	−0.116	0,275
										(Constant)	1.252	0.280		4.822	0.019

*Mean curve fit for linear, cubic and quadratic curves for dyads with 10 sessions. R square represents the proportion of the variance for movements synchrony per dyad explained by the number of sessions as the independent variable in the regression model. Adj R-square adjusts the statistic based on the number of independent variables in the model. Std. Error of Est. is the estimated standard deviation of an estimate measuring the uncertainty associated with the estimate. Standard errors are calculated from observed data. Sum of squares measures how far individual measurements are from the mean. df indicates the number of degrees of freedom of values that are free to vary and is defined as the minimum number of independent coordinates that can specify the position of the system completely. Mean square is the mean squared error and shows how close a regression line is to a set of points. F statistic is a value in ANOVA to find out if the means between two populations are significantly different. Sig./p-value indicates statistical significance and refers to the claim that a result from data generated by the experimentation is not likely to occur randomly. Unstand B is the unstandardized beta which represents the slope of the line between movement synchrony per dyad and thenumber of sessions. Unstand. CoStE represents the average distance that the observed values deviate from the regression line. Standardized Coef. Std. Error represents the standard error and measures the precision of the estimate of the coefficient. Stand. Coef. Beta indicates estimates resulting from the regression analysis where the underlying data have been standardized so that the variances of the number of sessions and movement synchrony per dyad are equal to 1. t is the t-statistic which represents the ratio of the departure of the estimated value of a parameter from its hypothesized value to its standard error*.

**Figure 2:**
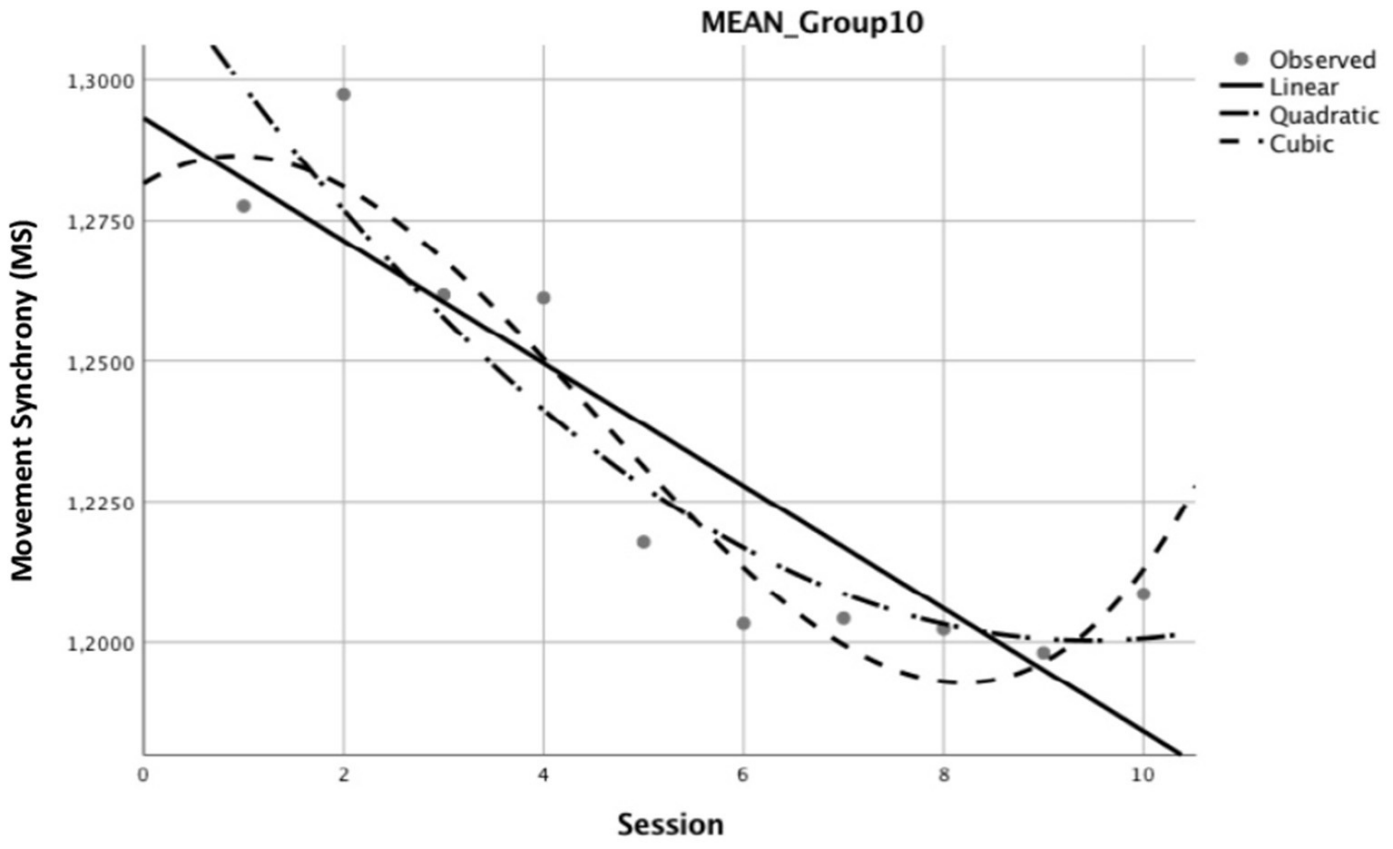
Best curve fit related to mean movement synchrony. Reduced best fitting curves for Mean Synchrony of dyads (*N* = 59) that completed coaching sessions represented in linear, quadratic, and cubic curves. The y-axis depicts the index of MS calculated as an expression of the movement coordination between coach and client.

Figure 2 depicts the graphical representation of the best curve fit in relation to mean MS for each of the three curve types (linear, cubic, and quadratic) after curve type reduction. It shows that all curves decrease (mean MS at the last session much lower than at the 1st session); some show a small “recovery” at the end (small increase in mean MS).

Correspondingly, Supplementary Table 3 details the correlation coefficients for these three best fitting curves in relation to mean MS. While the “linear decrease” curve type (*n* = 24) is the most frequently represented pattern of change of MS (data can be obtained upon request), it is not the best fit for the mean change in MS. The “cubic” curve type is the one that best explains mean variance in MS (R2 = 0.987) by the number of sessions.

#### Best Fitting Curves for 173 Dyadic Interactions

Supplementary Tables 4–6 explored best fitting curves for 173 dyadic interactions and corroborate the best-curve-fit estimations identified in the dataset of 59 dyads that completed 10 sessions.

**Figure 3:**
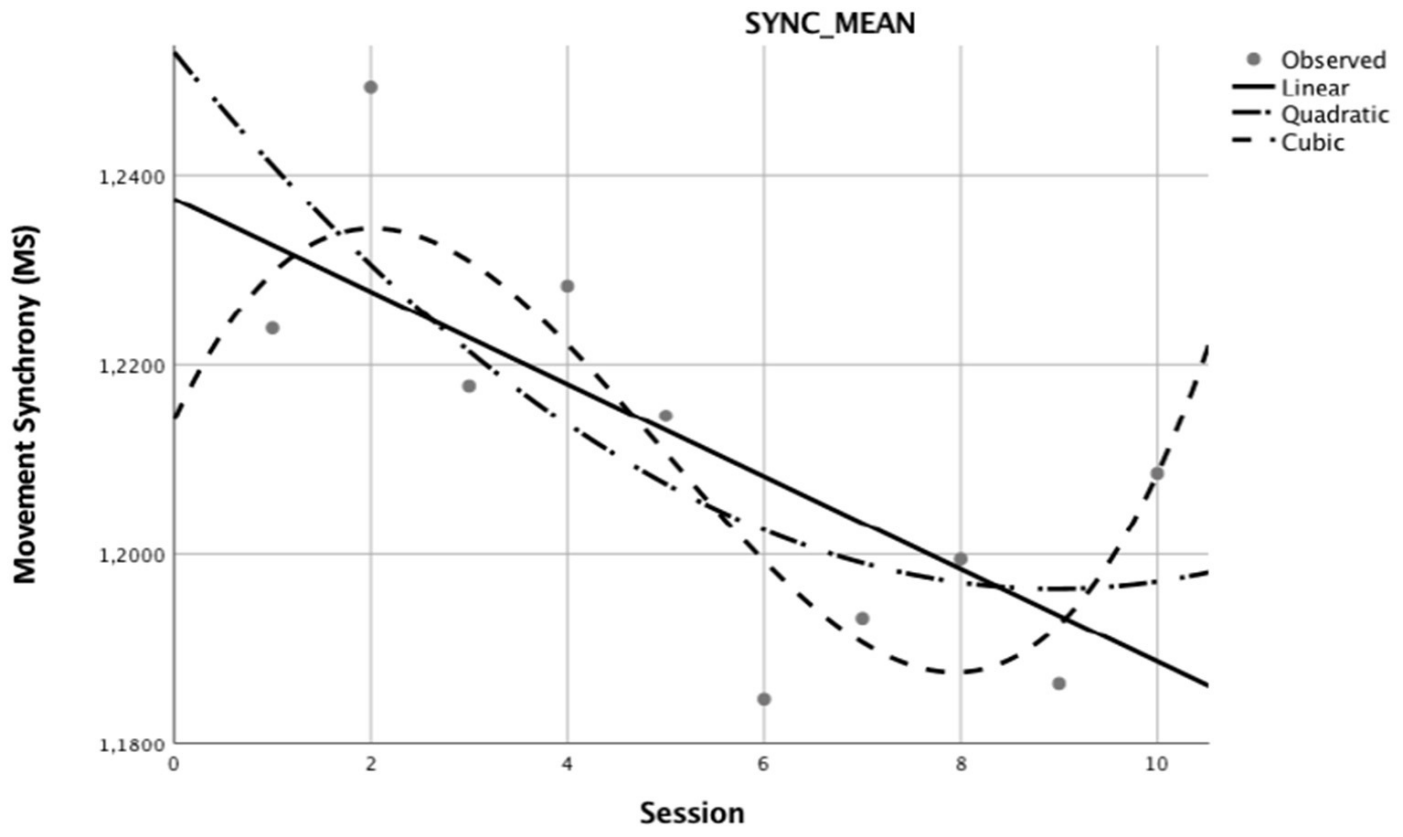
Mean best curve fit related to movement synchrony. Reduced best fitting curves for Mean Synchrony of dyads (*N* = 173) that completed coaching sessions represented in linear, quadratic, and cubic curves. The x-axis depicts sessions 1 to 10. The y-axis depicts the index of MS calculated as an expression of the movement coordination between coach and client.

Figure 3 depicts the graphical representation of the mean best curve fit in relation to MS for each of the three curve types (linear, cubic, and quadratic) after curve type reduction. Correspondingly, Supplementary Table 7 details the overall correlation coefficients for these three best fitting curves in relation to mean MS. Overall, the linear curve type (*n* = 79) is the most frequently represented pattern of change of MS but not the best fit for the mean change in MS. The “cubic” curve type is the one that best explains mean variance in MS (R2 = 0.763) by the number of sessions.

#### Linear Regression Analysis

A linear regression analysis was run for the dataset of 173 dyadic interactions where the curve type parameters (b or beta) were used as the dependent variable, and the demographic factors obtained from the pre-coaching questionnaire, as well as the number of sessions, were used as independent variables (Table 4). Additionally, interaction coefficients were calculated between each of these variables to ascertain the association between each best fit curve type [i.e., linear (b), quadratic (b^2^) or cubic (b^3^) best curve fit estimations] and the independent variables. SPSS V.25 was used for the linear regression analysis. The analysis showed low R^2^ of below.1 and, therefore, low goodness of fit (Table 4). However, R^2^ values lower than.5 are typical in any field that attempts to predict human behavior.

**Table 4:** Model fit with demographics and no of sessions.

	**Best fit curve types model fit with demographics and no of sessions—all dyads**											
	**Model summary**							**ANOVA**				
**Curve**	**R square**	**Adj R square**	**Std. error**	**R square change**	**F Change**	**sig. f change**		**Sum of**	**df**	**Mean square**	**F**	**Sig./**
			**of Est**.					**squares**				***p*** **-value**
Linear	0.080ª	−0.009	0.516	0.080	0.901	0.0565	Regression	3.604	15	0.240	0.901	0.565ªª
							Residual	41.616	156	0.267		
Quadratic	0.044ª	−0.048	0.178	0.044	0.475	0.950	Regression	0.225	15	0.015	0.475	0.950ªª
							Residual	4.933	156	0.032		
Cubic	0.045ª	−0.047	0.019	0.045	0.487	0.945	Regression	0.003	15	0.000	0.487	0.945ªª
							Residual	0.059	156	0.000		

*Linear, cubic and quadratic curves for all dyad interactions. R square represents the proportion of the variance for movements synchrony per dyad explained by the independent variables in the regression model. Adj R-square adjusts the statistic based on the number of independent variables in the model. Std. Error of Est. is the estimated standard deviation of an estimate measuring the uncertainty associated with the estimate. Standard errors are calculated from observed data. R Square Change is just the improvement in R-square when the second predictor is added. The R-square change is tested with an F-test, which is referred to as the F-change. A Sig. F-change means that the variables added in that step signficantly improved the prediction. Sum of squares measures how far individual measurements are from the mean. df indicates the number of degrees of freedom of values that are free to vary and is defined as the minimum number of independent coordinates that can specify the position of the system completely. Mean square is the mean squared error and shows how close a regression line is to a set of points. F statistic is a value in ANOVA to find out if the means between two populations are significantly different. Sig./p-value indicates statistical significance and refers to the claim that a result from data generated by the experimentation is not likely to occur randomly ª. Depdendent variable: linear, quadratic or cubic curve fit. ªª Predictors: (Constant), Experiance#Group, Gender_Coach#Gender_Client, ClientAge, Gender_Coach, Experiance, Gender_Coach#Group, Gender_Client#Experiance, Gender_Client#Group, ClientAge#Group, ClientAge#Gender_Client, ClientAge#Experiance, Gender_Coach#Experiance, ClientAge#Gender_Coach, Gender_Client, Group*.

First, analyzing the between-person (across dyads) variance in b's for across-session patterns in mean MS, we found that the across-session pattern of change in mean MS was not predicted by either independent variables *per se* or by interactions of the independent variables, indicating that these variables are not related to variance in MS across dyads. Second, only client age and coach years of experience were found to have a small but significant correlation with each other (r = 0.21, 2-tailed *p* = 0.01). Therefore, the demographic variables and the number of sessions operationalized in our study are not considered to be multicollinear.

In effect, b for the linear curve parameter is positive for the interaction between client age and coach experience, indicating an increase in the MS curve (Table 5a). We found similar indications in association with b^3^ for the cubic curve parameter (Table 5b), while b^2^ for the quadratic curve parameter was negative, indicating a weaker quadratic downward trend when client age and coach experience increase (Table 5c).

**Table 5a:** Linear curve type interactions.

**Linear curve type interactions with demographics and no of sessions**							
			**Coefficients**				
			**Unstandardized coefficients**		**Standardized coefficients**		
**Interaction variable**			**B**	**Coeff. St. Error**	**Beta**	***t***	**Sig**.
Client Age			−0.027	0.028	−0.541	−0.946	0.346
Gender_Coach			−0.611	0.796	−0.445	−0.767	0.444
Gender_Client			0.627	0.616	0.584	1.018	0.310
Coach_Experience			−0.392	0.649	−0.378	−0.604	0.547
Number of Sessions			0.004	0.123	0.020	0.033	0.947
ClientAge#GenderCoach			0.008	0.011	0.399	0.706	0.481
ClientAge#GenderClient			0.002	0.009	0.081	0.180	0.858
ClientAge#CoachExperience			0.018	0.008	1.009	2.166	0.032
ClientAge#Number of Sessions			−0.002	0.001	−0.485	−1.179	0.240
GenderCoach#GenderClient			0.025	0.247	0.054	0.101	0.919
GenderCoach#CoachExperience			−0.012	0.243	−0.026	−0.051	0.959
GenderCoach#Number of Sessions			0.035	0.043	0.376	0.796	0.427
GenderClient#CoachExperience			−0.279	0.188	−0.601	−1.490	0.138
GenderClient#Number of Sessions			−0.026	0.035	−0.292	−0.747	0.456
CoachExperience#Number of Sessions			0.021	0.033	0.237	0.626	0.532

*Sig./p-value indicates statistical significance and refers to the claim that a result from data generated by the experimentation is not likely to occur randomly. Unstand B is the unstandardized beta which represents the slope of the line between movement synchrony per dyad and the interaction variables. Unstand. CoStE represents the average distance that the observed values deviate from the regression line. Standardized Coef. Std. Error represents the standard error and measures the precision of the estimate of the coefficient. Stand. Coef. Beta indicates estimates resulting from the regression analysis where the underlying data have been standardized so that the variances of the number of sessions and movement synchrony per dyad are equal to 1. t is the t-statistic which represents the ratio of the departure of the estimated value of a parameter from its hypothesized value to its standard error*.

**Table 5b:** Quadratic curve type interactions.

**Quadratic curve type interactions with demographics and no of sessions**							
			**Coefficients**				
			**Unstandardized coefficients**		**Standardized coefficients**		
**Interaction variable**			**B**	**Coeff. St. Error**	**Beta**	***t***	**Sig**.
Client Age			0.008	0.010	0.492	0.844	0.400
Gender_Coach			0.107	0.274	0.232	0.392	0.696
Gender_Client			−0.118	0.212	−0.325	−0.555	0.579
Coach_Experience			0.145	0.224	0.415	0.649	0.517
Number of sessios			−0.023	0.042	−0.349	−0.550	0.583
ClientAge#GenderCoach			−0.002	0.004	−0.291	−0.506	0.613
ClientAge#GenderClient			−0.001	0.003	−0.141	−0.305	0.761
ClientAge#CoachExperience			−0.005	0.003	−0.891	−1.877	0.062
ClientAge#Number of Sessions			0.001	0.001	0.444	1.058	0.292
GenderCoach#GenderClient			0.013	0.085	0.083	0.152	0.879
GenderCoach#CoachExperience			−0.008	0.084	−0.050	−0.096	0.923
GenderCoach#Number of Sessions			−0.004	0.015	−0.125	−0.260	0.795
GenderClient#CoachExperience			0.047	0.065	0.297	0.722	0.471
GenderClient#Number of Sessions			0.004	0.012	0.147	0.370	0.712
CoachExperience#Number of Sessions			0.000	0.012	0.004	0.010	0.992

*Sig./p-value indicates statistical significance and refers to the claim that a result from data generated by the experimentation is not likely to occur randomly. Unstand B is the unstandardized beta which represents the slope of the line between movement synchrony per dyad and the interaction variables. Unstand. CoStE represents the average distance that the observed values deviate from the regression line. Standardized Coef. Std. Error represents the standard error and measures the precision of the estimate of the coefficient. Stand. Coef. Beta indicates estimates resulting from the regression analysis where the underlying data have been standardized so that the variances of the ineraction variables and movement synchrony per dyad are equal to 1. t is the t-statistic which represents the ratio of the departure of the estimated value of a parameter from its hypothesized value to its standard error*.

**Table 5c:** Cubic curve type interactions.

**Cubic curve type interactions with demographics and no of sessions**							
			**Coefficients**				
			**Unstandardized coefficients**		**Standardized coefficients**		
**Interaction variable**			**B**	**Coeff. St. Error**	**Beta**	***t***	**Sig**.
Client Age			−0.001	0.001	−0.577	−0.991	0.323
Gender_Coach			−0.009	0.030	−0.170	−0.288	0.774
Gender_Client			−0.004	0.023	−0.100	−0.171	0.864
Coach_Experience			−0.018	0.024	−0.468	−0.733	0.465
Number of sessios			0.002	0.005	0.304	0.480	0.632
ClientAge#GenderCoach			0.000	0.000	0.260	0.452	0.652
ClientAge#GenderClient			0.000	0.000	0.159	0.345	0.730
ClientAge#CoachExperience			0.001	0.000	0.871	1.835	0.068
ClientAge#Number of Sessions			−4.093	0.000	−0.307	−0.731	0.466
GenderCoach#GenderClient			−0.002	0.009	−0.127	−0.233	0.316
GenderCoach#CoachExperience			0.002	0.009	0.107	0.207	0.836
GenderCoach#Number of Sessions			1.784	0.002	0.005	0.011	0.991
GenderClient#CoachExperience			−0.001	0.007	−0.046	−0.112	0.911
GenderClient#Number of Sessions			0.001	0.001	0.211	0.531	0.596
CoachExperience#Number of Sessions			−0.001	0.001	−0.201	−0.521	0.603

*Sig./p-value indicates statistical significance and refers to the claim that a result from data generated by the experimentation is not likely to occur randomly. Unstand B is the unstandardized beta which represents the slope of the line between movement synchrony per dyad and the interaction variables. Unstand. CoStE represents the average distance that the observed values deviate from the regression line. Standardized Coef. Std. Error represents the standard error and measures the precision of the estimate of the coefficient. Stand. Coef. Beta indicates estimates resulting from the regression analysis where the underlying data have been standardized so that the variances of the interaction variables and movement synchrony per dyad are equal to 1. t is the t-statistic which represents the ratio of the departure of the estimated value of a parameter from its hypothesized value to its standard error*.

Finally, relating the positive and negative interactions for linear (b), quadratic (b^2^) or cubic (b^3^) parameters (Tables 5a–c) to best fit curves for mean MS comprising all the 173 dyads in Supplementary Table 7, we found that (a) the higher the client and coach experience, the more the MS curve increases significantly even when, overall, the MS curve shows a clear downward trend across sessions for all 173 dyads; (b) the quadratic downward trend is less strong when client age and coach experience increase, albeit less significantly, as reflected in the overall MS curve trend for all 173 dyads, and (c) the higher the client age and coach experience, the more the MS curve increases, albeit insignificantly, even when, overall, the MS curve shows first an upward, and then a downward and then, again, an upward trend for all 173 dyads.

## Discussion

As a complementary investigation to the study by Erdös and Ramseyer (2021), this study represents the first attempt to (1) describe the dynamic MS pattern within coach-client dyads across sessions and (2) link that pattern to several demographic predictors and the number of coaching sessions. Our multiple-step exploratory study resulted in two key findings: first, we report the downward trend in the reduced best fitting curve types (linear, quadratic, and cubic) for mean MS in (a) the dyads that completed 10 sessions, and (b) all 173 dyads with varied numbers of sessions. Second, regression analysis comprising 173 dyads to explore the strength of relationship between curve type parameters and the demographics selected in the present study revealed that client age and coach years of experience show a small but significant correlation.

### A Differential Downward Trend of Movement Synchrony

While the downward trend for MS is clearly indicated for both clusters (i.e., 59 dyads that completed 10 sessions, and 173 dyads that completed varied numbers of sessions), the downward trends indicate a differential change in MS over time. The difference is indicated in that the cubic curve type suggests a slight increase in MS both at the outset of the coaching engagement and at the end of the coaching trajectory. This difference is more clearly indicated in the cluster that involved 173 dyads. While the higher level of MS at the outset of coaching engagements was associated with coaches' efforts to “get on the same page with clients” (Erdös and Ramseyer, 2021, p. 15), the slight increase in MS indicated at the end of the trajectory is a nuanced pattern that this exploratory approach is the first to suggest in association with the length of the trajectory as well as in interaction with client age and coach experience. Earlier studies (e.g., Baron and Morin, 2009, 2010) purport that the number of sessions predicts higher client self-efficacy, and that this association is explained by the quality of the coach-client relationship. Recent studies (e.g., Sonesh et al., 2015) have reported that the number of sessions held is associated with client goal attainment, indicating that having 1–3 sessions is better than having 4–6 sessions but not as effective as having 7–9 sessions. Although the latter indication may appear counter-intuitive, it supports the nuanced time-series changes in MS that the cubic curve type suggests at the end of the coaching trajectory. It is possible that MS increases as some signal of change after 10 sessions again, but we need future research to further investigate the role of number of sessions in association with change in MS at the end of the coaching engagement.

Generally, neither the demographic factors nor the number of sessions discriminated among the groups of curve types. Additionally, the cluster of 173 dyads showed certain curve type variations in the change of MS in relation to the number of sessions completed (i.e., a downward trend is smaller with a cubic curve, showing clearer trends at the outset and at the end of the coaching trajectory). Therefore, we argue that process research needs to investigate MS (a) as an inherent relationship factor in association with other contextual factors such as the coaching theme or the gravity of the content dealt with in sessions, and (b) involve large samples of coach-client pairs with a minimum population size of 150 dyads and a coaching engagement of up to 10 sessions to arrive at clear deductions and a deeper understanding of the role of MS in the coach-client relationship.

Specifically, regarding the slight increase in MS reflected by the cubic curve type variation, Erdös and Ramseyer (2021) discussed that higher MS at the outset with a subsequent decrease in MS can be interpreted as a “correctional mechanism” (p. 15) where a coach and a client perceive the coach-client relationship as yet unstable. In this vein, the slight increase in MS found at the end of coaching in this study may imply a similar phenomenon. We propose that a coach and a client sync in with each other more on completion of the coaching again as the coach-client relationship will feel “disrupted” or in some way incomplete to them. Therefore, we recommend future research to look into the temporal dynamics of MS as reflected by the cubic curve type as a way of “getting stuck/unstuck.” Such relational dynamics at the outset and end of the coaching are likely to yield insight into how MS is affected in a client's goal pursuit.

As goes for the decrease in MS based on curve type variations, we argue that those variations imply nuanced relational dynamics in coaching and propose three interpretations. First, the dyadic stage at which a coach and a client succeed and fail in meeting each other's needs differs in the goal-attainment process. Second, the curve-type variations may indicate that MS becomes more or less relevant in the coach-client relationship at different points in time. Indeed, other factors such as a client's stage of autonomy or maturity may determine how the change process develops for each dyad at a different stage. Third, clients may grow their self-regulatory capacities in resolving presenting issues each at a different moment. The stage at which they may feel less impacted by a coach's decreased level of spontaneous responsiveness to their needs will differ in each dyad. Conversely, the stage at which coaches may grow more daring and risk taking as clients grow more autonomous in how they address own challenging issues will differ in each dyad too. Hence, while MS as a signal of embodied congruence between a coach and a client at the outset of coaching may become less necessary for feeling safe later on in the process, this decrease will depend on the nuanced relational dynamics of each dyad. Eventually, the coach and the client can allow themselves to “make mistakes” without either the client or the coach feeling impacted by any “ugly” situation engendered by lack of spontaneous responsiveness to their needs, depending on their dyadic progress.

These three interpretations find support in some studies in development sciences (e.g., Tronick and andBeeghly, 2011), which report that synchrony as a set of interactional exchanges represents alternating periods of dynamic patterns of matching, mismatching, or reparation, potentially as a purposeful meaning making. Tronick and andBeeghly, 2011 argue that mutual regulation through higher initial synchronous interactional exchanges between infants and caregivers is likely to repair the infant-caregiver relationship in the face of the caregiver's failure to spontaneously respond to the infant's needs at a later point in time. Furthermore, as clients' autonomy to reach goals on their own has been found to be a key element of goal attainment (Schiemann et al., 2018), we recommend coaching research to further investigate the predictive value of client autonomy for goal attainment through MS across varied number of sessions. This investigative approach may be an additional gateway to how we can conceptualize the importance of embodied congruence as an interpersonal phenomenon in presence-based coaching in the future.

Conclusively, our study is the first to answer some scholars' call (e.g., Jackson, 2017) that we need exploratory studies to establish useful hypotheses of non-verbal dynamics in coaching for qualitative and quantitative research, and that coaching research needs new methodological solutions beyond coach and client self-reports (i.e., automated measurement tools) when tapping into non-verbal interactions to develop the coaching evidence base.

### Regression Analyses

Generally, the variation in predictor variables, which were selected on a thorough theoretical basis, was not found to be associated with the variation in patterns of change in mean MS across sessions and dyads. This finding prompts us to ask if there is anything to predict at all. Potentially, variations are contained within the process of coaching and cannot be determined, nor can they be eliminated. All the more, as the model fit calculations for best curve types in association with interaction patterns of predictor variables produced different results too, on the one hand, in the set of 59 dyads that completed 10 sessions, we found no significant associations between interaction patterns and the model fit for best curve types, although there is a clear linear downward trend as shown in Figure 2. On the other hand, in the set of all 173 dyads, we found positive interaction patterns for client age and coach experience for the linear downward trend in MS, although the best model fit showed that the cubic curve type was most suitable.

Specifically, our exploratory investigations showed (a) no positive correlations between the number of sessions and change in MS, and (b) only client age and coach years of experience showed a small but significant association with linear MS dynamics.

On the one hand, some coaching literature reports similar results when studying coaching outcomes in association with the number of sessions. Other studies report differential results, as discussed below. One meta-analysis (Theeboom et al., 2014) examining the effects of the number of sessions on the overall longevity of coaching interventions found that a greater number of sessions did not relate to higher effectiveness of coaching. However, that meta-analysis tested only for linear effects of the number of sessions on the longevity of coaching and outcomes. Another meta-analysis on the effectiveness of workplace coaching (Jones et al., 2014) tested the number of sessions (coaching schedule) for moderation, including linear and curvilinear effects in their analysis. That meta-analysis concluded that none of the effects were significant, and that neither longevity of the coaching intervention (in weeks) nor the number of sessions moderated coaching outcomes. In particular, curvilinear effects indicated that there was a plateauing of the impact of coaching. That indication may mean that additional sessions do not impact up and that even short-term coaching may have a beneficial impact on client goal attainment. Most recently, some coaching effectiveness outcome studies (de Haan et al., 2019; Molyn et al., 2019; Zimmermann and Antoni, 2020) have found that, while the effectiveness of coaching grew over the course of the coaching engagement as an overall measure, the level of effectiveness in terms of outcomes changed from session to session. This may be the result of each consecutive session in the coaching engagement, having its own specific challenges (and, therefore, levels of effectiveness in terms of outcomes). This indication is corroborated in studies investigating dynamic change processes (i.e., forming, norming, storming, performing phases) in team development (e.g., Kozlowski and Chao, 2018), which report that team-level processes and outcomes are multilevel phenomena that emerge and bottom up from the interactions among team members over time, under the shifting requirements of each work context. In one particular coaching study (Molyn et al., 2019), only some process variables such as the task-focus component of working alliance predicted outcomes when measured at the outset and if regressed against the last session outcome. In-between sessions, only task-focus predicted a coaching outcome, and this task-outcome effect was not found to be produced for each data point.

On the other hand, there are a few studies conducted on interactional processes in coaching (Schermuly and Scholl, 2012; Ianiro et al., 2013, 2015; Ianiro and Kauffeld, 2014), which report that dominant-friendly interactional behaviors between a coach and a client in the first session lead to a client's feeling safe in the relationship after the third session. Yet, the association between MS and the number of coaching sessions has remained unaddressed in coaching process research. In this context, it is noteworthy that, while non-verbal literature shows that females exhibit more engaging behaviors in unstructured conversations, which includes synchrony (e.g., Fujiwara et al., 2019), this study cannot report any significant mixed gender effects of MS, which may be due to coaching involving more structured conversations. Both female coaches and clients exhibited high MS, while coaching sessions had a more standardized nature (see Table 6 for coach selection criteria).

**Table 6:** Frequency distribution of sample by country.

	**Frequency distribution**		
**Country**	**Frequency**	**Valid percent**	**Cumulative percent**
Australia	7	3.8	3.8
Austria	2	1.1	4.9
Belgium	4	2.2	7.4
Brazil	4	2.3	9.2
Canada	3	1.6	10.9
Chile	2	1.1	12.0
China	2	1.1	13.0
Czech Republic	4	2.2	15.2
Denmark	2	1.1	16.3
Ecuador	4	2.2	18.5
Egypt	2	1.1	19.6
Finland	2	1.1	20.7
France	1	0.5	21.2
Greece	9	4.9	26.1
Hungary	2	1.1	27.2
India	5	2.7	29.9
Indonesia	4	2.2	32.1
Ireland	2	1.1	33.2
Italy	4	2.2	35.3
Japan	2	1.1	36.4
Kazakhstan	2	1.1	37.5
Lithuania	2	1.1	39.2
Netherlands	22	12	50.5
Poland	3	1.6	52.2
Romania	2	1.1	53.3
Saudi Arabia	21	11.4	64.7
Singapore	1	0.5	65.2
Slovenia	4	2.2	67.4
South Africa	3	1.6	69
South Korea	2	1.1	70.1
United Kingdom	35	19	89.1
USA	20	10.9	100
Total	184	100	

*Frequency indicates the number of participants per country. The Valid Percent column shows the percentage that does not include missing cases. Cumulative Percent adds the percentages of each region from the top of the table to the bottom, culminating in 100*.

As regards to the interaction effects about client age and coach experience, while some studies show that gender similarity (e.g., Bozer et al., 2015) has (a small) significant effect on a coaching outcome, there is less convergence in findings when it comes to coach's attributes such as coaching experience. For instance, Lai and McDowall (2014) report that coach training/background has a significant influence on the coaching process and results, while Sonesh et al. (2015) showed that the coach's experience was unrelated to client goal attainment. We argue that the emerging lack of convergence in coaching literature implies that we may only interpret findings appropriately if and where we sufficiently account for the context in which coaching has taken place, and that it may, therefore, be inappropriate to draw simple generalized conclusions.

### Conceptual Considerations

In this study, MS is conceptualized as a relational embodied phenomenon. Non-verbal responses through the body as they occur spontaneously in interaction with an interlocutor beyond conscious awareness are argued to play the central role in coaching. The body is viewed as the instrument that will indicate the extent to which interlocutors are congruent with each other at any given moment. This conceptualization implies that the coach's “way of being present with clients” has the potential to shape the clients' ways of being present with the coach. Hence, in our study, Silsbee's (2008) suggestion that the body is able to work in partnership is consistent with how we conceptualize congruence as a reciprocal exchange expressed through the coach's and the client's non-verbal responses to coaching. Silsbee (2008) purports that this partnership ultimately means that presence evokes change in both the coach and the client rather than the client alone. Hence, we propose that presence is not about the coach alone. It works both ways in a nuanced dynamic way. A client's spontaneous non-verbal responses will equally evoke change in the coach. This proposition reflects the theory of interpersonal movement coordination (IMC, Bernieri and Rosenthal, 1991), which claims that the quality of how two individuals manage to spontaneously respond to each other at the moment will influence the affective and mental states of both interactants in the relationship. Furthermore, we argue that, unless clients perceive the coach as congruent in how the coach responds to their needs beyond the use of verbal language, the clients will not experience rapport, trust or empathy (Kolden et al., 2011), which are crucial components of working alliance (Bordin, 1979).

Hence, relational embodied congruence as conceptualized in this study possibly plays an important role in how presence as a key element of coaching effectiveness (e.g., Jackson, 2017) can emerge in the coach-client relationship. Jackson (2017) argues that, by developing a greater understanding of physicality as an embodied perspective on coaching, we can gain an understanding of felt experiences as subtle practices in coaching (Gendlin, 2003; Stelter, 2021), which mark third-generation coaching (Stelter, 2014) today.

### Theoretical Implications

While the study by Erdös and Ramseyer (2021) observed the downward trend in MS in a general manner, it did so informally as the focus of that paper was on the role of MS as a predictor of goal attainment as moderated by working alliance in workplace coaching over time. In contrast, this study provided an in-depth exploration of the downward trend in MS, producing a series of curve type trends. While the overlaps and differences between the two studies have been extensively discussed, this section adds the theoretical implications of the differential findings in terms of the cubic curve type. It is the one that best explained mean variance in MS by the number of sessions. This curve type also suggested a slight increase in MS both at the outset of the coaching engagement and at the end of the coaching trajectory. The difference was more clearly indicated in the cluster that involved 173 dyads. Theoretically, this curve type can be connected to a third dimension. This is because cubic curves can be used to model phenomena in three dimensions, allowing for the possibility to identify a missing dimension or explore the result of changes to one or more dimensions. Therefore, the findings of this study on the cubic curve type substantially imply that MS is a multidimensional interpersonal phenomenon and that the differential effects of MS may only be shown clearly on the basis of a larger population. Furthermore, we theorize that only curve type variations can bring to light the dynamic nature of dyadic processes in coaching. Consequently, we argue that MS needs to be researched as a change process involving more interactional and interpersonal process variables on the basis of large population numbers if we are to contribute to a holistic theory-building design in coaching (Myers, 2017).

### Recommendations for Coaching Research

First, given the unexplored within-dyadic change of MS across time segments in our study and based on our conceptual considerations, we recommend coaching process research to further investigate the following themes:

1. studying the development of MS dynamics of a total coach-client trajectory within a session to explore synchrony dynamics in the first session as compared to synchrony dynamics in the final session for that dyadic trajectory;
2. studying the change patterns in MS of all coach-client trajectories within a session to study synchrony dynamics in all first sessions as compared to synchrony dynamics in all final sessions for the entire set of dyadic trajectories.

Second, as we found no association between the differential downward trend of MS and demographic factors and number of sessions, we advocate fresh approaches to research on MS based on the following questions:

1. How is the differential decrease of MS related to working alliance in coaching?
2. What is the role of embodied congruence as some expression of authentic interpersonal exchange for coaching success?

Both questions warrant investigation into coaching as embodied congruence has remained a coach-specific concept of presence (Silsbee, 2008). Hence, we urge scholars to investigate congruence as an authentic interpersonal exchange between a coach and a client—that is, as an expression of relational presence—for coaching success.

Finally, as goes for the small but significant correlation between client age and coach years of experience, we recommend coaching research to specifically investigate these parameters in association with MS and embodied congruence in randomized controlled trials where there is possibility to control for these variables.

### Recommendations for Coaching Practice

We recommend coaches to be trained in working with MS as a dynamic interactional and interpersonal phenomenon as it is shown to have a differential relational impact on a client's progress in workplace coaching: It is always both. In doing so, coaches may grow a capacity to discern moments of “stuckness” through non-verbal interactional processes in the coaching engagement. This is particularly relevant as MS as authentic embodied congruence can be applied to practice third-generation coaching (Stelter, 2014).

### Limitations

The most important limitation of the present exploratory approach is that it is not a randomized control trial as the most optimal design (Robertson et al., 2017). It is an ex-post exploration without accounting for a waitlist control group.

## Conclusions

The exploratory approach investigating the question “What is in the trajectory of dyadic interactions?” in association with MS in coaching as a change process indicates that there is a differential downward trend in MS across sessions over time. This indication has substantial theoretical implications for conceptualizing interactional processes in coaching. Hence, we need further studies in coaching process research to deepen our understanding of the predictive impact of the differential downward trend in MS on a client's autonomy beyond coaching engagements.

## Data Availability Statement

The original contributions presented in the study are included in the article/Supplementary Material, further inquiries can be directed to the corresponding author/s.

## Ethics Statement

The studies involving human participants were reviewed and approved by ABRI Amsterdam Business Research Institute at Vrije Universiteit Amsterdam, The Netherlands. The patients/participants provided their written informed consent to participate in this study.

## Author Contributions

TE: conducting original research, data administration and ethics handling, designing methods and analysing data, conceptualizing and theorizing for the introduction section, writing up the discussion section, and handling the reference list. PJ: peer support for the drafting of the manuscript and providing the four-eye principle for the data analysis section to ensure it bears a high-level quality mark for publication. Both authors contributed to the article and approved the submitted version.

## Conflict of Interest

The authors declare that the research was conducted in the absence of any commercial or financial relationships that could be construed as a potential conflict of interest.

## Publisher's Note

All claims expressed in this article are solely those of the authors and do not necessarily represent those of their affiliated organizations, or those of the publisher, the editors and the reviewers. Any product that may be evaluated in this article, or claim that may be made by its manufacturer, is not guaranteed or endorsed by the publisher.
